# In vivo investigation of ear canal pulse oximetry during hypothermia

**DOI:** 10.1007/s10877-017-9975-4

**Published:** 2017-01-27

**Authors:** K Budidha, P A Kyriacou

**Affiliations:** Research Centre for Biomedical Engineering, School of Mathematics, Computer Science & Engineering, City, University of London, Northampton Square, London, EC1V 0HB UK

**Keywords:** Ear canal, Hypothermia, Photoplethysmography, Pulse oximetry

## Abstract

Pulse oximeters rely on the technique of photoplethysmography (PPG) to estimate arterial oxygen saturation (SpO$_2$). In conditions of poor peripheral perfusion such as hypotension, hypothermia, and vasoconstriction, the PPG signals detected are often weak and noisy, or in some cases unobtainable. Hence, pulse oximeters produce erroneous SpO$_2$ readings in these circumstances. The problem arises as most commercial pulse oximeter probes are designed to be attached to peripheral sites such as the finger or toe, which are easily affected by vasoconstriction. In order to overcome this problem, the ear canal was investigated as an alternative site for measuring reliable SpO$_2$ on the hypothesis that blood flow to this central site is preferentially preserved. A novel miniature ear canal PPG sensor was developed along with a state of the art PPG processing unit to investigate PPG measurements from the bottom surface of the ear canal. An in vivo study was carried out in 15 healthy volunteers to validate the developed technology. In this comparative study, red and infrared PPGs were acquired from the ear canal and the finger of the volunteers, whilst they were undergoing artificially induced hypothermia by means of cold exposure (10 $^\circ$C). Normalised Pulse Amplitude (NPA) and SpO$_2$ was calculated from the PPG signals acquired from the ear canal and the finger. Good quality baseline PPG signals with high signal-to-noise ratio were obtained from both the PPG sensors. During cold exposure, significant differences were observed in the NPA of the finger PPGs. The mean NPA of the red and infrared PPGs from the finger have dropped by >80%. Contrary to the finger, the mean NPA of red and infrared ear canal PPGs had dropped only by 0.2 and 13% respectively. The SpO$_2$s estimated from the finger sensor have dropped below 90% in five volunteers (failure) by the end of the cold exposure. The ear canal sensor, on the other hand, had only failed in one volunteer. These results strongly suggest that the ear canal may be used as a suitable alternative site for monitoring PPGs and arterial blood oxygen saturation at times were peripheral perfusion is compromised.

## Introduction

A pulse oximeter is a non-invasive optical device used to provide a continuous and robust measure of arterial oxygen saturation (SpO$_2$). The device measures SpO$_2$ by shining light at two different wavelengths into the vascular tissue (such as the finger or the ear lobe) and sensing the changes in light absorption of the oxygenated and deoxygenated haemoglobin produced during arterial pulsations [1]. The device has since its invention in the 1970s revolutionised anaesthesia and critical care. The popularity of the device and its increased clinical use in recent years has driven the manufacturers and researchers to consistently develop its hardware, software and signal processing algorithms. However, there still remain a few unresolved problems that limit its performance. Possibly, the most important limitation of the device in its current state is the inability to estimate accurate SpO$_2$ in conditions of poor peripheral perfusion. Poor perfusion can result from various clinical conditions such as hypotension [2, 3], hypothermia, vasoconstriction [4], low cardiac output [5], and peripheral vascular disease. These clinical situations, can occur in patients undergoing major surgical procedures such as cardiopulmonary bypass surgery or in patients with chronic cardiovascular complications and renal failure [6]. The SpO$_2$ readings in these conditions may become very inaccurate or cease altogether. The failure of the device in these circumstances is directly associated with the inability of the pulse oximeter probe, placed at the periphery (finger or toe) to detect adequate photoplethysmographic signals which are necessary for the estimation of SpO$_2$ by pulse oximetry.

Many attempts were made previously to minimise or eliminate this limitation by the application of sensors on better-perfused areas such as the forehead [7], nose [8] and oesophagus [9]. These sensors, however, experience functional difficulties such as attachment problems, venous pulsations, and motion artefacts [7]. In some cases, the sensors are semi-invasive and require considerable expertise for use in clinical practice. Thus, SpO$_2$ readings are still unobtainable or inaccurate at just the time when they will be most necessary. Hence, the ear canal was proposed as a possible site for reliable monitoring of PPG signals and SpO$_2$. The hypothesis underlying this choice was that the ear canal, being closer to the trunk, and being supplied by the arteries that supply blood to the brain, would remain adequately perfused during low perfusion states. Also, the anatomy of the external ear canal would provide a natural anchoring for the sensor. Although the same hypothesis was applied in developing in-ear heart rate monitors by companies such as Bargi and Cosinuss$^o$, the ear canal has not been explored for SpO$_2$ measurements. The only attempt to measure SpO$_2$ from the tissue surrounding the ear canal was by venema et al, who have developed an in-ear sensor to measure SpO$_2$ from the targus of the ear [10]. However, so far they have not presented any investigations into the morphology or the quality of PPG signals that can be acquired during hypothermia.

Hence, a reflection based, dual wavelength ear canal PPG probe was developed along with a PPG processing system. The feasibility of measuring PPGs and SpO$_2$ from the ear canal and its performance in conditions of locally induced peripheral vasoconstriction (right-hand immersion in ice water) was previously tested in 15 volunteers [11]. However, the cold pressor test described in [11] has only validated the ear canal sensor’s performance in states of local hypothermia. To truly show the potential of this site for SpO$_2$ and PPG signal monitoring, it is necessary to test the sensor in more natural conditions leading up to hypothermia. Hence, it is proposed that the developed technology be tested in healthy volunteers undergoing whole body cold exposure as it results in heat loss from all portions of the body and stimulates more natural responses a patient would experience in conditions leading up to hypothermia. This paper describes the proposed technology in brief and illustrates the effects of body cooling on the acquired PPGs and SpO$_2$ measured from the finger as opposed to the new ear canal sensor.

## Methods and materials

### Measurement setup

**Fig. 1:**
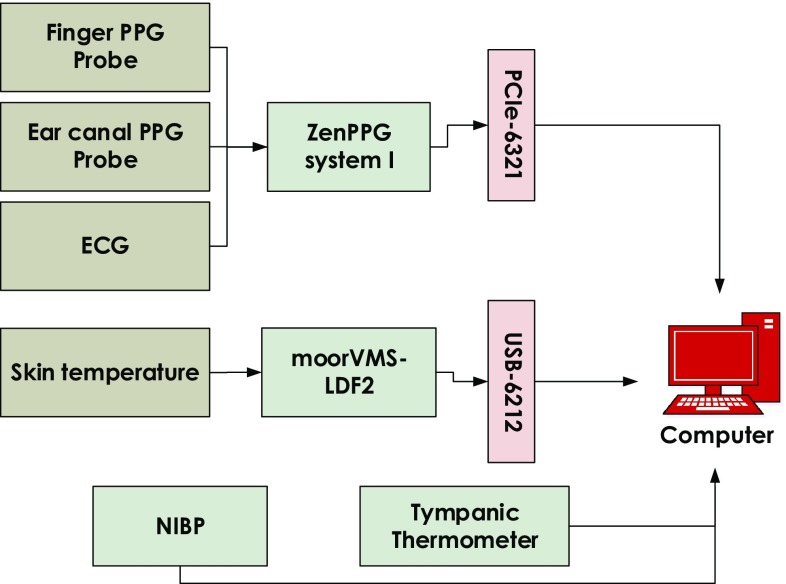
Block diagram showing the measurement setup used during the cold exposure test. PPG, ECG, and temperature signals were acquired using the setup

The measurement setup was designed to simultaneously detect, sample, record and display PPG, ECG and temperature signals. The block diagram of the entire measurement system is shown in Fig. 1. The system consists of the following.

#### PPG sensors

**Fig. 2:**
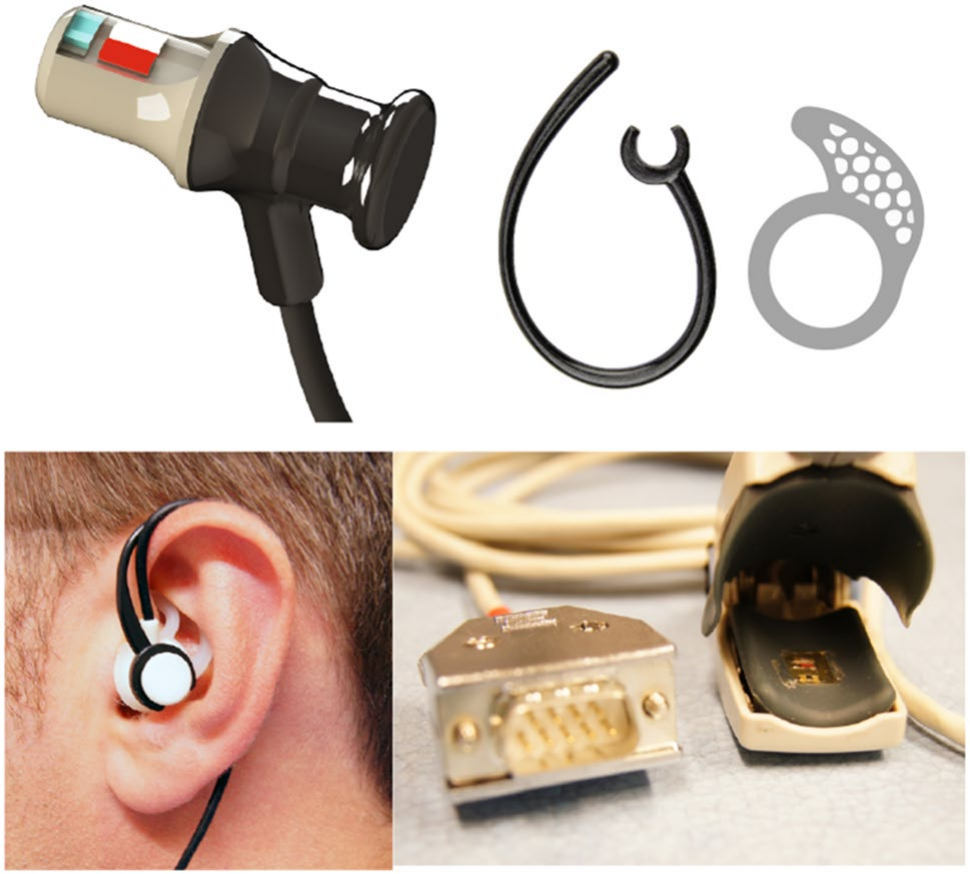
A 3D sketch of ear canal PPG sensor, photograph of an assembled ear canal PPG sensor placed inside the right ear of a volunteer and a photograph of the finger sensor

The ear canal PPG probe is an earphone shaped reflectance PPG probe consisting of two surface mount LEDs and a photodiode. The LEDs used to emit light at 870 nm in the infrared region and 658 nm in the red region *(CR 50 IRH and CR 50 1M, Excelitas technologies, Massachusetts, USA)*. The photodetector used is a flattop photodiode with an active area of 0.65 mm$^2$ and peak sensitivity at 900 nm *(SR 10 BP-BH, Excelitas technologies, Massachusetts, USA)*. The LEDs and the photodiode were placed 5 mm apart from each other as experimental studies have previously shown that a separation of 4 to 5 mm yields a better signal-to-noise ratio [12]. The sensor was designed such that, PPG signals can be acquired from the bottom surface of the outer ear canal (Fig 2). The ear canal probe was manufactured using the FORMIGA P-110 SLS 3D printer (EOS – Electro-Optical Systems, Munich, Germany). The biocompatible material used to manufacture the probe case was Feinpolyamid PA-2200 (Nylon) (EOS - Electro-Optical Systems, Munich, Germany). The sensor also consisted of an ear hook that anchored on top of the helix and a silicone ear fin that fit inside the concha of the ear to hold the probe in place and reduce motion artefacts. The part of the sensor that fit inside the ear canal has an overall diameter of 7 mm.

A reflectance finger PPG probe, optically identical to the ear canal probe was also developed to facilitate comparisons of SpO$_2$ measured from the ear canal and the finger. The finger probe was encapsulated within a conventional pulse oximeter clip. In order to avoid direct contact between the optical components and the skin, all sensors were sealed using medical graded clear epoxy resin *(DYMAX 141-M, Dymax Corporation, Torrington, CT)*.

#### Instrumentation

The raw PPG signals (AC + DC) from both the ear canal and the finger were acquired using a modular, dual channel, dual wavelength PPG processing system named ZenPPG [13]. The system consisted of the circuitry required for intermittent switching of light sources, the independent sampling of red/infrared PPG signals, preconditioning and outputting the acquired signals to a data acquisition system. The system also incorporated a three lead ECG amplifier for monitoring the R-waves of the QRS complex of the ECG signal.

A laser Doppler flowmeter *(moorVMS-LDF2, Moor Instruments, Devon, U.K.)* was used to measure peripheral skin temperature and flux (indirect blood flow measure). However, the flux measurements are not used in this paper. Along with the LDF, a commercial non-invasive blood pressure (NIBP) monitor was used to measure the systolic and diastolic blood pressure *(HEM-907, Omron Healthcare, Hoofddorp, The Netherlands)*. A tympanic thermometer *(ThermoScan-5 IRT4520, Braun GmbH, Frankfurt, Germany)* was used to measure the core temperature from the ear canal of the volunteer.

#### Data acquisition

The red and infrared PPG signals acquired from the ear canal and the finger were digitised and recorded along with the ECG signal using a PCIe-6321 NI DAQ cards *(National Instrument Corporation, Austin, Texas)*. An NI USB-6212–bus powered USB DAQ card was used to record the skin temperature signals from the LDF. The simultaneous acquisition of all signals and control of the systems was through a virtual instrument (VI) implemented in LabVIEW. All the signals were recorded at a sampling frequency of 1 kHz. The blood pressure reading and core temperatures were recorded manually in an Excel sheet during the study.

### Subjects

Following the approval from the Senate Research Ethics Committee of the City University London, fifteen healthy volunteers (6—female  and 9—male) aged between 19 and 45 (mean age ± SD—28 ± 5 years) were recruited for this study. Based on their medical history, volunteers with cardiovascular, pulmonary, or metabolic diseases were excluded from the study. Before the study, heart rate, blood pressure and core body temperature were recorded for each volunteer. All the subjects were found to be normotensive (mean BP ± standard deviation (SD)—116/70 ± 14/11), normothermic (mean core temp ± SD—36.52 ± 0.33) and none was taking any medication. The subjects were informed of the details of the study and a signed informed consent was sought from all the volunteers before the experiment. All the subjects were asked to refrain from ingesting beverages containing caffeine and alcohol and were asked not to exercise or smoke for at least two hours preceding the test. To maximise the effect of cold temperatures on the cardiovascular system, all the subjects were asked to wear just one layer of clothing during the experiment.

### Measurement protocol

The trials were carried out in the Biomedical Engineering Research Laboratory, at City University London. Upon arrival, all the volunteers were seated in a room maintained at $24\pm 1^{\circ }$C for a minimum of 10 min to ensure haemodynamic stabilisation. During the study, the subjects were sited in a comfortable chair, with both hands resting on the armrests arranged to a height approximately equivalent to their heart’s position. Once the volunteer was comfortable, heart rate (HR), blood pressure and core temperature were measured. If the volunteer was found to be normotensive and normothermic then, the study was continued. The following sensors were then attached to the volunteer–

- The finger PPG probe was placed on the second digit of the left hand, and the ear canal PPG sensor was placed 9 mm inside the left ear canal of the volunteer
- The LDF sensor was placed just below the thumb on the dorsal surface of the left hand and it was attached to the skin by means of a ring-shaped double-sided adhesive
- The red, yellow and green leads of the ECG cable were connected to the Ag-AgCl easitab ECG electrodes (SKINTACT, F-WA00) placed directly on the chest (the right and the left side) and on the left hip.

Once all the sensors were in place, the investigation protocol started with the acquisition of baseline measurements from the volunteer for at least 2 min. The volunteers were then moved to the adjacent temperature-controlled room maintained at $10\pm 1\,^{\circ }$C for 10 min. After the cold exposure, the volunteers were moved back to normal room temperatures ($24\,^{\circ }$C), where monitoring continued for another 10 min. PPG, ECG and temperature data was continuously recorded during all three phases of the experiment. The core temperature was measured from the right ear of the volunteer once every minute for the entire duration of the study (22 min). Blood pressure was intermittently measured from the right arm once at the start of the study, and then at the end of the cold exposure and the recovery period.

### Data analysis

The raw PPG, ECG and temperature data recorded during the study were extracted separately for offline analysis. Prior to any signal processing, the acquired signals were resampled to 100 Hz. This was to restrict the bandwidth of the signals and remove unwanted noise. The resampled signals were then filtered and processed to calculate various parameters, described as follows:

*Normalised Pulse Amplitude (NPA)* — The red and infrared PPG signals acquired from both locations were first separated into AC and DC components using bandpass and lowpass filters implemented in LabChart–8.0 *(AD Instruments, Sydney, Australia)*. The lower and upper cut-off frequencies of the bandpass filter (AC signal) were 0.5 and 15 Hz respectively. The cut-off frequency of the lowpass filter (DC signal) was 0.5 Hz. The filters used were linear-phase Finite Impulse Response (FIR) filters with a transition width of 20% and pass band ripple <0.5% (the input amplitude). The effective length of the lowpass and bandpass FIR filters was 139 and 3980 respectively. The AC component of the PPG signals was then divided by the DC component to normalise the PPG signals.

A peak detection algorithm was used to detect the peaks and valleys of all the normalised PPG signals. From the detected peaks, the normalised pulse amplitude (NPA) was calculated. The mean NPA estimated from the red and infrared PPG signals acquired from both the ear canal and the finger was then averaged for every two minutes of the study. NPA was estimated by the above procedure for all the subjects and was then averaged for the entire group. The mean NPA of the study group during cold exposure (every 2 min) and recovery (every 2 min) periods was analysed for statistical significance compared to the baseline period. A non-parametric statistical test (ANOVA on ranks) was performed on the data. A P-value <0.05 was considered to be statistically significant. Statistical analysis was performed using SigmaPlot-12.0 (SPSS Inc, Chicago, USA).

*SpO* $_2$ *analysis*—In order to demonstrate the effect of compromised peripheral perfusion on the estimation of arterial oxygen saturation, SpO$_2$ values were calculated from the PPG signals acquired from both the ear canal and the finger probes during all three phases of the experiment. SpO$_2$ was calculated in a three-seconds rolling window using Eq. (1).

1 $$\begin{aligned} SpO_2 = 110 - 25 \times R_{OS}; \qquad R_{OS} = \frac{\left( \frac{AC_R}{DC_R}\right) }{\left( \frac{AC_{IR}}{DC_{IR}}\right) } \end{aligned}$$

where $R_{OS}$ is the Ratio of Ratios, $AC_{IR}$ and $AC_R$ are the peak-to-peak amplitudes of the infrared and red AC PPGs, and $DC_{IR}$ and $DC_R$ are the DC PPG components at respective wavelengths. The SpO$_2$ estimated from each volunteer was averaged for every two minutes of the study. The mean SpO$_2$ estimated during the first 2 min (i.e., baseline) was then compared with every 2 min (2–10) of the cold exposure and the recovery periods. Further, the change in SpO$_2$ as a response to the cold exposure was calculated, and the number of instances where the SpO$_2$ value has dropped below 90% was computed and considered as a failure. The failure rates of both sensors were then compared.

## Results

**Fig. 3:**
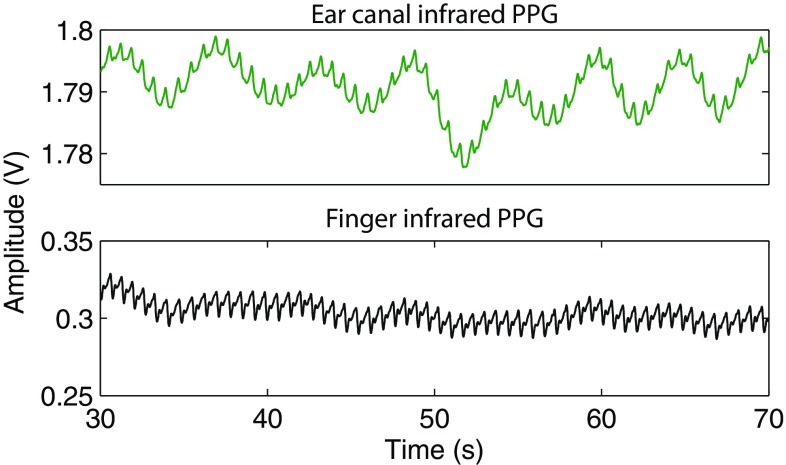
The raw infrared PPG signals acquired from the ear canal (*green trace*) and the finger (*black trace*) of a volunteer

Good quality, easily recognisable raw PPG signals with large amplitudes and high signal to noise ratio were recorded from the ear canal and the finger of all the volunteers. Figure 3 depicts baseline raw (AC + DC) infrared PPG signals acquired from the ear canal and the finger of a volunteer. Two key observations can be made from the figure—(1) the pronounced respiratory modulation in the ear canal PPG signals when compared to the finger PPGs, and (2) the large DC amplitude and small AC amplitude of the ear canal PPG signals when compared to the PPG signals acquired from the periphery. The disparity in the amplitude of the PPG signals is expected as the tissue lining the ear canal (2–3  mm) is much smaller than the finger (10–15 mm). Hence, the light absorption by tissue and other non-pulsatile absorbers is much higher in the finger than that of the ear canal.

**Fig. 4:**
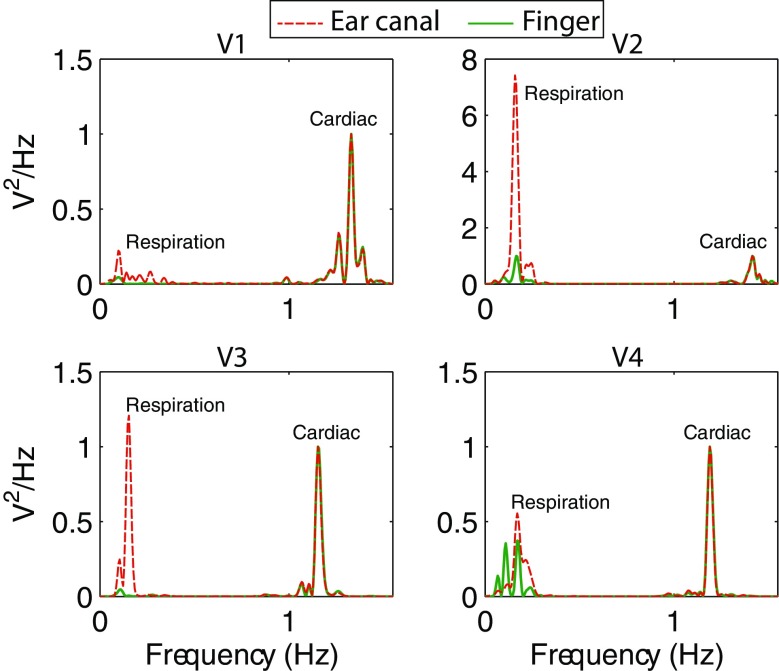
The power spectrum of the infrared raw PPG signals acquired from the ear canal and the finger of four healthy volunteers, highlighting the respiratory and cardiac components. The power of the respiratory component in the EC PPG signals is significantly higher in all subjects

The disparity in the respiratory modulation of the ear canal and finger PPG signals was further investigated by taking the power spectrum of the raw PPG signals. The power spectrum of the baseline raw infrared PPGs acquired from both the locations in four healthy volunteers is shown in Fig. 4. In this figure, the power spectrum of the ear canal and the finger PPG signals were normalised with the power of the cardiac component to highlight just the changes in the respiration related component. It is evident that the power of the respiration related frequency component is much higher in the ear canal PPG signals than that of the periphery. Similar results were demonstrated in the work carried out by Shelley et al in [14]. Two factors are likely to contribute to these findings. First, the shorter distance of the ear canal from the heart compared to the finger, which meant less attenuation of the respiratory modulation. Second, the blood vessels in the head region are relatively less sensitive to sympathetically mediated vasoconstriction that may mask respiratory oscillations.

### AC PPG signals

**Fig. 5:**
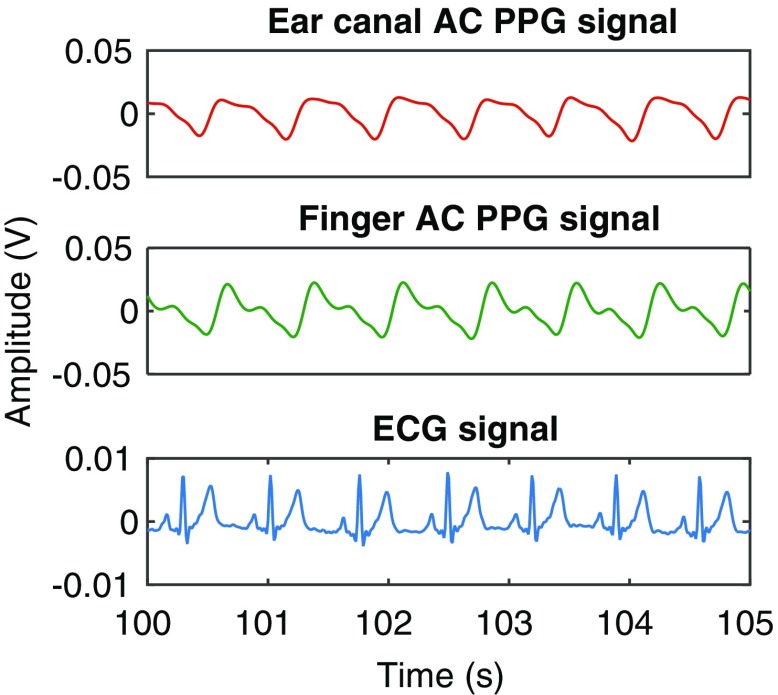
The infrared AC PPG signals acquired from the ear canal and the finger, and the simultaneously acquired ECG signal from one of the volunteers

A 5-s sample of the baseline infrared AC PPG signals acquired from the ear canal and the finger of one of the volunteers (Volunteer 6) is shown in Fig. 5, along with the simultaneously acquired ECG signal. As can be observed from the figure, the morphology of the PPG signal acquired from the ear canal was distinct from the finger PPG signal. These changes in the morphology of the PPG signals are thought to be due to the vascular resistance of large arteries that supply blood to the head and the brain (common carotid arteries). Similar morphological differences were observed in the PPG signals acquired from most volunteers. Irrespective of these differences, the PPG signals acquired from both locations were synchronous with the R-wave peak of the ECG signal.

**Fig. 6:**
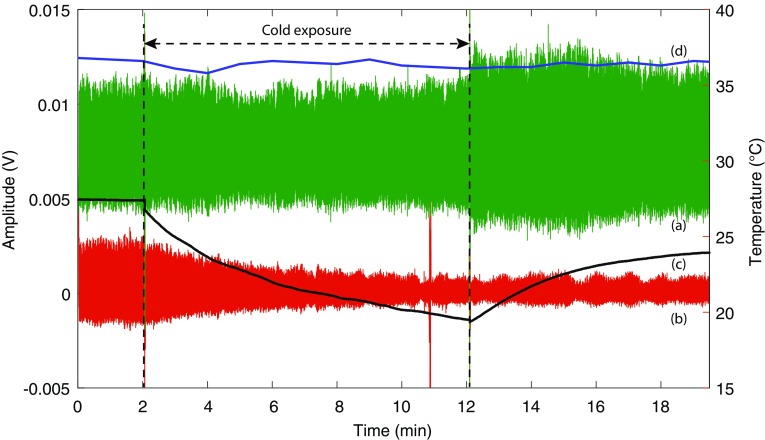
Infrared AC PPG signals acquired from (*a*) the ear canal, and (*b*) the finger of a volunteer and the simultaneously acquired (*c*) skin and (*d*) core temperature signal for the entire duration of the study. The Y-axis on the left shows the amplitude of the PPG signals while the Y-axis on the right shows the temperature. The spike in finger PPG signal at 11th min is a movement artefact

Figure 6 shows the infrared PPG signals acquired from the finger and the ear canal along with the peripheral (skin) and the core temperature measured from the same volunteer for the entire duration of the study (22 min). From the figure, it is evident that the amplitude of the PPG signals acquired from the finger has reduced significantly with time during cold exposure. This was, however, expected due to the profound vasoconstriction resulting from exposure to low temperatures. The skin temperature of the volunteer has dropped from 27.4 ± 0.02$\,^\circ$C during baseline to 20.2$\,^\circ$C by the end of the cold exposure. On the other hand, the amplitude of the PPG signals from the ear canal has remained relatively constant throughout the cold exposure. The maximum drop in the core temperature of the volunteer during the cold stimulus was only 0.9$\,^\circ$C, which is below the ±1$\,^\circ$C error of the digital tympanic thermometer. This explains the high amplitude PPGs acquired from the ear canal during the cold exposure. The blood pressure of the volunteer was increased from 110/60 to 119/71 by the end of the cold exposure due to vasoconstriction.

During the recovery period, the amplitude of the PPG signals acquired from both locations have increased with an increase in skin temperature. However, the amplitude of the finger PPG signals did not return to the initial baseline value within the 10 min recovery period. The ear canal PPG signals, on the contrary, have increased in amplitude as soon as the volunteer was removed from the air-conditioned room, and have remained relatively constant for the rest of the monitoring period. The skin and the core temperature of the volunteer by the end of the recovery period were 24.1$\,^\circ$C and 36.8$\,^\circ$C respectively. The blood pressure has also recovered back to 111/60 by the end of the study. Most volunteers in the study group had a similar response to this volunteer during the cold exposure. To analyse these changes further, and to take into account the changes in the DC portions of the PPG signals, NPA (AC/DC) of the PPG signals was measured.

### Normalised pulse amplitude (NPA)

**Fig. 7:**
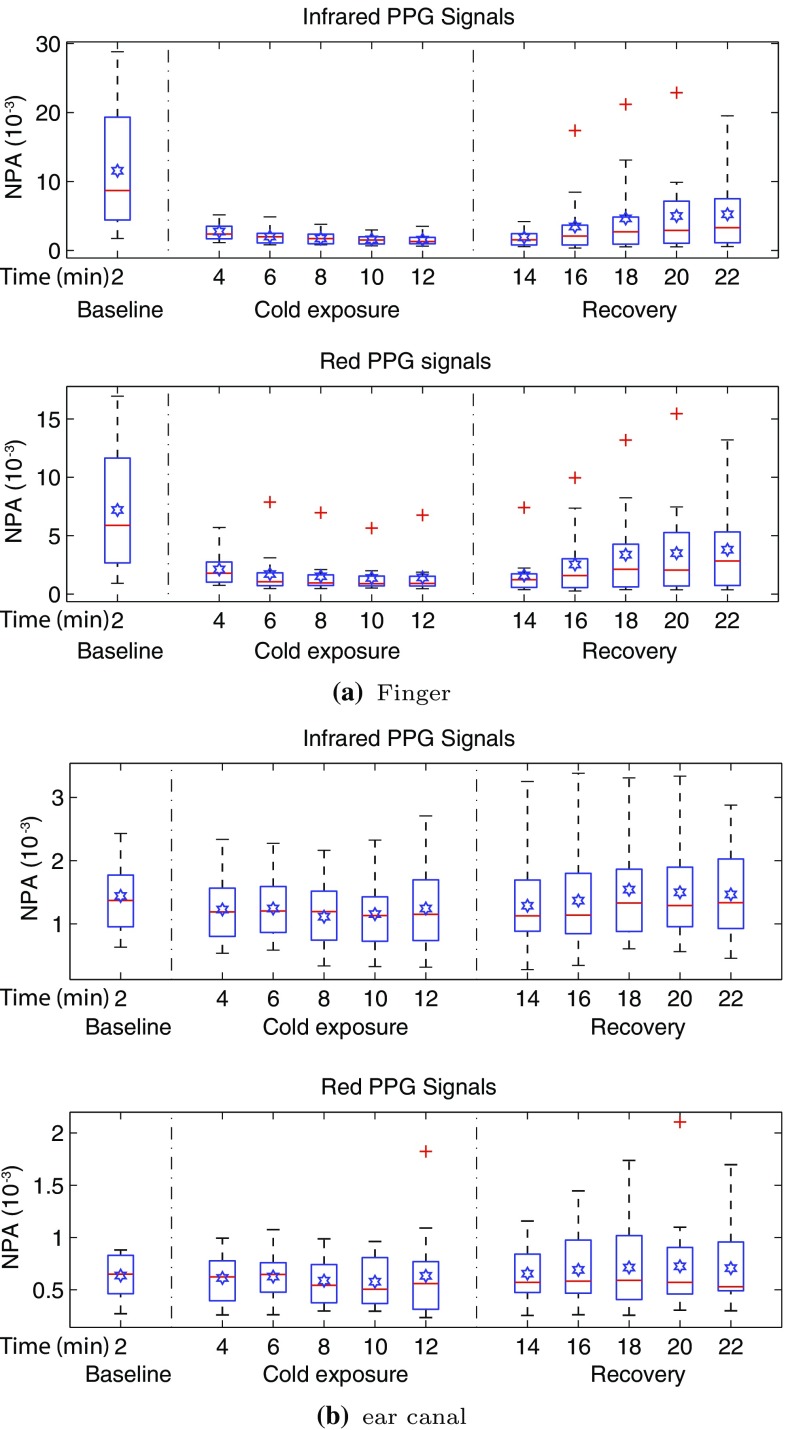
NPA of **a** the finger and **b** the ear canal PPG signals acquired from 15 volunteers during all three stages of the experiment. Each box shows the mean NPA measured across a 2 min period in all the volunteers. The *red line* in each box shows the median value of the data, the  shows the mean, and $+$ shows the outliers

The NPA of the red and infrared PPG signals acquired from both the finger and the ear canal was measured and averaged for every two minutes of the study in all the volunteers. The distribution of this data is graphically displayed using the Box and Whiskers plots in Fig. 7.

The sudden exposure to cold temperatures during the experiment caused an instantaneous and significant drop in the mean NPA of red and infrared finger PPG signals in all volunteers. This is evident through the significant reduction in the interquartile range, the mean and the median at the 4th min in Fig. 7a, when compared to the first 2 min. The NPA of the finger PPG signals had further reduced with time across the volunteer group at a steady rate until the end of the cold exposure. During the recovery period, the NPA of the finger PPG signals slowly increased with time. However, the NPA did not rise to a value close to the baseline, although a steady state was achieved between the 20th and 22nd min. In contrast with the finger, the NPA measured from the ear canal did not change during the cold exposure. As seen in Fig. 7b, the interquartile range, the mean and the median have all remained relatively constant throughout the experiment.

**Table 1:** Summary of the statistical test results obtained from the Kruskal-Wallis test performed on the NPA of red and infrared PPG signals acquired from the finger, and the ear canal of the volunteers

Location	P-values		H-value		Statistical significance
	Red	Infrared	Red	Infrared	
Finger	=0.002	<0.001	28.05	35.67	Yes
Ear canal	=0.993	=0.847	2.38	5.61	No

A P-value <0.05 indicates a statistically significant difference. The highest H-value corresponds to the largest discrepancy between rank sums

To check if there were any statistically significant differences between the NPA of red and infrared PPG signals measured during baseline, cold exposure and the recovery periods, statistical analysis was performed on the measured data. Prior to the statistical tests, the normality of the data was tested using the Kolmogorov-Smirnov test with Lilliefors’ correction. As not all the data was found to be normally distributed, it was decided that a non-parametric test will be used. The test used was Kruskal-Wallis One Way Analysis of Variance on Ranks. The mean NPA of all the subjects during baseline (every 2 min) was compared with every 2 min mean of the cold exposure and the recovery periods for statistical significance (i.e., baseline vs. 4 $\rightarrow$ 22 min, total of ten comparisons per PPG signal). The summary of the results of the Kruskal-Wallis test is presented in Table 1. Statistically significant (p < 0.05) differences were found between all the groups when NPA of red and infrared finger PPG signals was compared during the study. No significant difference was found between any of the groups when NPA of the ear canal was compared during the study.

During the cold exposure, the mean skin temperature of the volunteers has dropped to 19.5 ± 0.49$\,^\circ$C (± standard error of mean (SEM)) from 29.9 ± 0.42$\,^\circ$C during baseline. The mean core temperature of the volunteers has remained unchanged (baseline: 36.6 ± 0.07$\,^\circ$C, cold: 36.0 ± 0.11$\,^\circ$C). This conceivably explains the uncompromised blood flow to the ear canal and, therefore, the unwavering PPG signals obtained from the ear canal. The BP of the volunteers has increased from 115/79 ± 3.6/2.7 during baseline to 125/75 ± 4.2/2.9 during cold exposure.

### Arterial oxygen saturation (SpO$_2$)

**Fig. 8:**
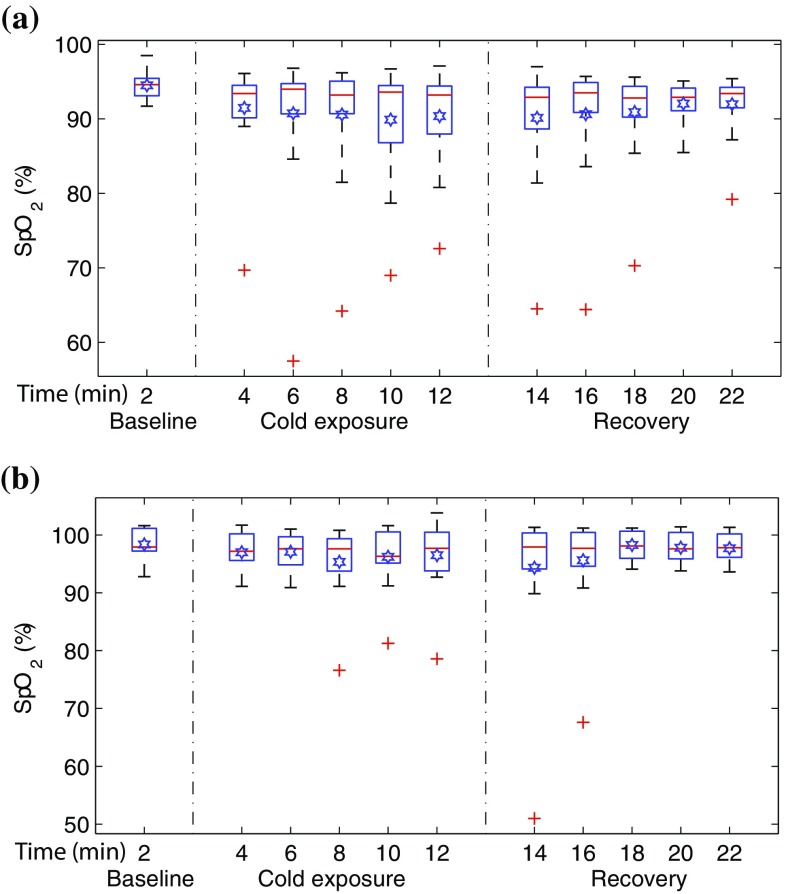
Box and whiskers plots demonstrating the change in mean SpO$_2$ measured for every two minutes of the study in **a** the finger and **b** the ear canal. SpO$_2$ estimated from the finger have dropped significantly towards the end of the study when compared to the ear canal. The *red line* in each box shows the median value of the data, the  shows the mean, and $+$ shows the outliers

To show the effect of the cold exposure on the acquired PPG signals and subsequently the effect on the SpO$_2$ estimated by the pulse oximeter, arterial oxygen saturation values were calculated from the PPG signals acquired from both sensors. The mean SpO$_2$ values calculated for every two minutes of the study in all the volunteers is displayed in Fig. 8 with the help of boxplots.

The SpO$_2$ values estimated from both the uncalibrated probes during baseline were in the healthy adult oxygen saturation range (94–100%). The mean SpO$_2$ (±SEM) calculated for the entire group during baseline was 95 ± 0.45% in the finger, and 98 ± 0.7% in the ear canal. During the cold exposure, the mean SpO$_2$ (represented by  in Fig. 8(a)) estimated from the finger probe had dropped with time, particularly in the last 4 minutes of the cold exposure. The mean finger SpO$_2$ (±SEM) of the volunteer group by the end of the cold exposure was 90 ± 1.6%. These low SpO$_2$ values would normally indicate hypoxia in clinical circumstances, and hence are inaccurate. During the recovery period, the mean SpO$_2$ estimated from the finger has slowly recovered with time (93 ± 2%). The mean SpO$_2$ (±SEM) calculated from the ear canal probe has remained relatively constant throughout the cold exposure (97 ± 1.5%) and recovery periods (98 ± 0.6%).

**Table 2:** Summary of the pulse oximeter failure during the cold exposure and the recovery period in 15 volunteers

No. of volunteers with SpO$_2$ < 90%											
Sensor	Cold exposure (min)						Recovery (min)				
	4	6	8	10	12		14	16	18	20	22
Finger	4	2	3	4	5		4	3	3	2	2
Ear canal	0	0	1	1	1		1	1	0	0	0

However, since blood oxygenation is a global variable which does not change in healthy volunteers from site to site, the differences observed in mean SpO$_2$ amongst the sensors was further investigated. To differentiate the volunteers in whom one of the pulse oximeters had failed (i.e., inaccurate SpO$_2$) from the other, the percentage drop in SpO$_2$ for every 2 min of the study was calculated in each volunteer. The number of instances in which the SpO$_2$ estimated from a particular probe had dropped to a value <90% was calculated and considered as a failure. Table 2 shows the number of volunteers in whom the estimated SpO$_2$ has dropped to a value <90% during the cold exposure and the recovery period. From the table, the finger probe produced erroneous SpO$_2$ readings in four volunteers as soon as they were exposed to cold air, and by the end of the cold stimulus, five volunteers had SpO$_2$ below the 90% mark. The ear canal pulse oximeter, on the other hand, had failed in one volunteer towards the end of the cold exposure.

**Fig. 9:**
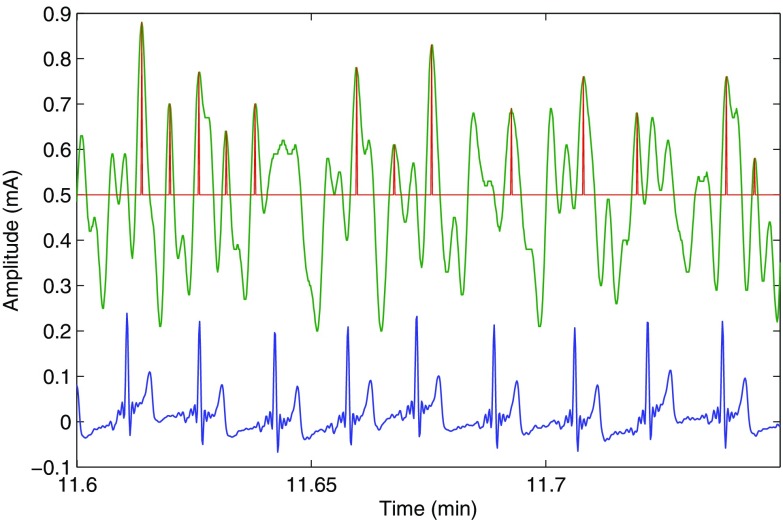
The red finger PPG signal (green trace) and the ECG signal (blue trace) acquired from a volunteer towards the end of the cold exposure, along with the peaks detected (red trace) by the peak detection algorithm. The amplitude of the* red* finger PPG signals was sometimes so weak that the peak detection algorithm could not distinguish between the heart beat peaks and the noise peaks

**Fig. 10:**
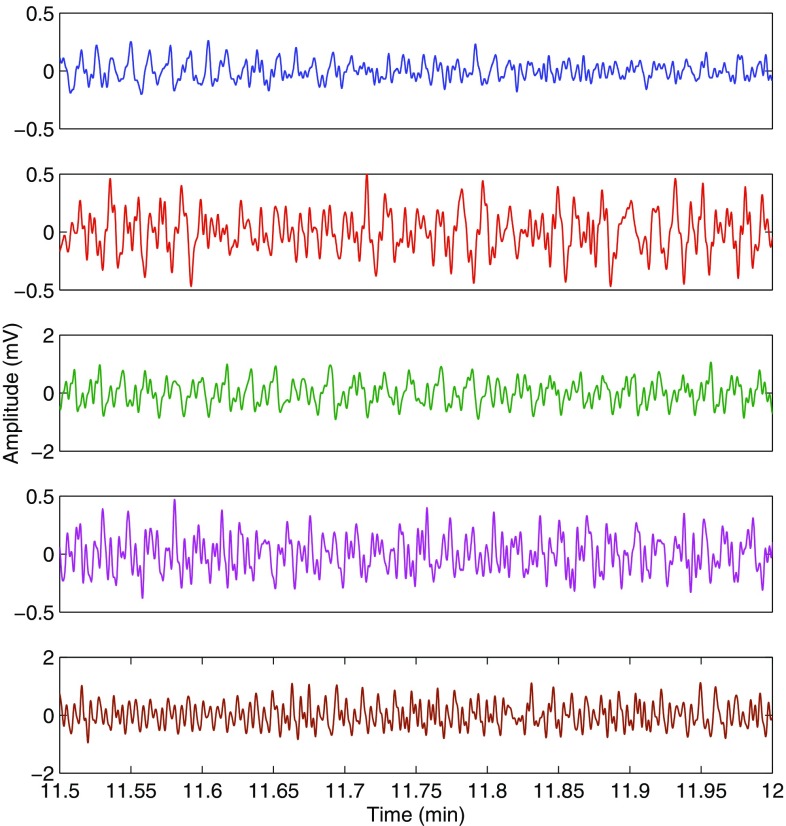
Red PPG signals acquired from the fingers of the volunteers in whom accurate estimation of SpO$_2$ was not possible during the cold exposure. The PPG signal is indistinguishable from noise in these cases

The high failure rate of the finger pulse oximeter is due to the very weak and noisy PPG signals recorded during the cold exposure. The peak detection algorithm in the pulse oximeter cannot distinguish between the heart pulses and the noise peaks in these situations, therefore producing inaccurate readings. To demonstrate this, the red finger AC PPG signal acquired from a volunteer during the cold exposure is shown in Fig. 9, along with the peaks detected by the algorithm and the ECG signal. The quality of the red finger PPG signals acquired from the five volunteers in whom accurate SpO$_2$ estimation was not possible is shown in Fig. 10. In this figure, arterial pulsations are unidentifiable in any of the PPG signals. Hence, the calculated SpO$_2$ readings were inaccurate.

## Discussion

Blood oxygen monitoring using peripheral pulse oximetry is susceptible to inaccuracies in conditions of compromised peripheral perfusion. In order to address this issue, the ear canal has been proposed as a new monitoring site for measuring PPG signals and SpO$_2$ on the hypothesis that, the ear canal will remain sufficiently perfused in states of low peripheral perfusion. To test this hypothesis a novel ear canal PPG sensor was developed along with an optically identical finger PPG probe and an acquisition system. The performance of the developed technology was tested in 15 healthy volunteers undergoing whole-body cooling. During the study, red and infrared PPG signals were acquired from the ear canal and the index finger of the volunteers experiencing cutaneous vasoconstriction by exposure to low temperatures (10$\,^\circ$C) for 10 min. The red and infrared PPG signals acquired from both the sensors during the study were then analysed and compared with respect to their shape, normalised amplitude and the SpO$_2$ estimated.

The PPG signals acquired from all the volunteers were generally of very good quality. The morphology of the PPG signals acquired from the ear canal were distinct from the finger PPG signals (Fig. 5). These changes in the morphology of the PPG signals are thought to be due to the vascular resistance of large arteries that supply blood to the head and the brain (common carotid arteries). A slight shift in phase between the finger and ear canal PPG signals is also apparent in Fig. 5. This is expected, as the ear being closer to the heart than the finger, the time taken for arterial pulsations to travel from the heart to the ear canal is less than the time is taken for the pulsations to reach the finger.

The NPA of the red and infrared PPG signals from the finger was significantly reduced as soon as the volunteer was exposed to cold temperature and has further decreased with time. Statistically, significant differences were found in the mean NPA of the finger PPG signals (P-value: red—0.002, infrared—<0.001) when the baseline measurement was compared with every 2 min mean of cold exposure. The mean percentage drop in the NPA of red and infrared finger PPG signals by the end of the cold exposure was 80.1 and 86.3% respectively. Statistical significant differences were also found when the baseline finger NPA was compared with every 2 min mean of the recovery period. This suggests that the NPA of red and infrared PPG signals has reduced significantly during the cold exposure and never recovered back to the baseline value in the monitoring period. The percentage difference between the mean NPA measured by the end of the study to the baseline was 47.2 (red) and 54.5% (infrared). These results demonstrate the sensitivity of the arterial vessels in the periphery to the vasoconstrictor stimuli.

In contrast with the finger, the NPA of the ear canal PPGs has remained relatively constant throughout the study. The mean drop in the red and infrared NPA of the ear canal PPGs was only 0.2 and 13% respectively. No significant difference was found (P-value: red—0.993, infrared—0.847) between any of the groups when the NPA of the ear canal PPGs from baseline was compared with cold exposure and recovery periods.

These disparities between the NPA of PPG signals acquired from the finger and the ear canal (or, in other words, the variations in the effect of cold exposure on the blood supply) are due to the thermal adaptation of the body. When the human body is exposed to cold, the efferent sympathetic nerves descending from the posterior hypothalamus (the body’s thermostat) produce intense constriction of the cutaneous blood vessels and closure of the arteriovenous anastomoses. This innervation reduces the heat transfer from the body’s core to the body’s surface and subsequently the heat loss to the environment but at the expense of further cooling the extremities [15, 16]. Hence, appendages such as the finger, which are part of the peripheral circulation are more affected by the cold than the central areas such as the ear canal. These results compliment and align well with the previously reported results by Awad et al in [17].

The SpO$_2$ estimated from both probes during baseline measurements was in the adult normoxic range (94–100%). However, during the cold exposure, the mean SpO$_2$ measured from the finger has dropped significantly with time, particularly in the last 4 min of the cold exposure. The mean SpO$_2$ calculated from the ear canal, on the other hand, has remained relatively constant throughout the study, with the exception of one outlier (see Fig. 8b). The increase in variability (interquartile range) of the finger SpO$_2$ values with time and the relatively stable median in Fig. 8a, however, indicates that the SpO$_2$ might have dropped significantly only in a few volunteers. Hence, the failure rate of each probe was quantified by calculating the number of volunteers in whom the estimated SpO$_2$ had dropped to a value <90%. The finger probe produced erroneous SpO$_2$ readings in a maximum of five volunteers during the cold exposure. The ear canal pulse oximeter, on the other hand, had failed only in one volunteer.

The high failure rate of the finger sensor during cold exposure was not due to the reduction in blood oxygenation but is merely due to the quality of the PPG signals acquired. When the body is exposed to ambient temperatures of 10$\,^\circ$C, the blood flow through the hand is minimal (less than 1 ml/min) [15, 18]. Hence, the PPG signals recorded in these situations are very weak and noisy. In some volunteers, the amplitude of the PPG signals diminished so significantly that the peak detection algorithms could not distinguish between noise and PPG signal peaks. Hence in these volunteers, the amplitude of the noise is being measured instead of the PPG signal. Figure 9 clearly demonstrates this effect. Since the amplitude of noise in both the red and infrared channels is approximately similar, the absorbance ratio (R$_{OS}$) will drive towards 1, resulting in a SpO$_2$ close to 85% (i.e., a failure). The quality of the red finger PPG signals acquired from the five volunteers in whom accurate SpO$_2$ estimation was not possible is shown in Fig. 10. As can be seen from the figure, it is very hard to differentiate the arterial pulsations from the noise in any of the five PPG signals. Hence the reason for inaccurate SpO$_2$ estimation by finger pulse oximeter.

In conclusion, it is fair to say that the SpO$_2$ measurements made from the finger are susceptible to poor peripheral perfusion and are heavily dependent on the thermal state of the site. This limitation really weakens the ability of the pulse oximeters in conditions when they are most needed. The newly developed ear canal probe on the other side offered reliable SpO$_2$ measurements even under the influence of the cold temperatures or profound vasoconstriction. However, more trials need to be conducted in more healthy volunteers and patients in order to assess this hypothesis.
